# Social determinants, health status and 10-year mortality among 10,906 older adults from the English longitudinal study of aging: the ATHLOS project

**DOI:** 10.1186/s12889-018-6288-6

**Published:** 2018-12-10

**Authors:** Natasa Kollia, Francisco Félix Caballero, Albert Sánchez-Niubó, Stefanos Tyrovolas, José Luis Ayuso-Mateos, Josep Maria Haro, Somnath Chatterji, Demosthenes B. Panagiotakos

**Affiliations:** 1 Department of Nutrition and Dietetics, School of Health Science and Education, Harokopio University, 70 Eleftheriou Venizelou Ave, 176 61 Attica, Athens Greece; 2 Department of Preventive Medicine, Public Health and Microbiology, Universidad Autónoma de Madrid, Madrid, Spain; 3 Instituto de Salud Carlos III, Centro de Investigación Biomédica en Red de Salud Mental, CIBERSAM, Monforte de Lemos 3-5. Pabellón 11, 28029 Madrid, Spain; 4 Hospital Universitario de La Princesa, Instituto de Investigación Sanitaria Princesa (IP), Madrid, Spain; 5 Parc Sanitari Sant Joan de Déu, Barcelona, Spain; 6 CIBER of Epidemiology and Public Health, Madrid, Spain; 7 Department of Health Metrics and Measurement, World Health Organization, Geneva, Switzerland

**Keywords:** Health, Healthy aging, Education, Financial status, Social determinants, Socioeconomic, Mortality

## Abstract

**Background:**

In either rich or poor countries, people’s health widely depends on the social conditions in which they live and work – the social determinants of health. The aim of the present work was to explore the association of educational and financial status with healthy aging and mortality.

**Methods:**

Data from the English Longitudinal Study of Aging (ELSA) were studied (*n* = 10,906 participants, 64 ± 11 years, 55% women). A set of 45 self-reported health items and measured tests were used to generate a latent health metric reflecting levels of functioning referred to as health metric (higher values indicated better health status). Overall mortality after 10-years of follow-up (2002–2012) was recorded.

**Results:**

Both education and household wealth over time were positively associated with the health metric (*p* < 0.001) and negatively with overall mortality (*p* < 0.001). Lifestyle behaviors (i.e., physical activity, smoking habits and alcohol consumption) mediated the effect of education and household wealth on the health metric and the latter mediated their effect on overall mortality.

**Conclusions:**

In conclusion, reducing socioeconomic disparities in health by improving the access to education and by providing financial opportunities should be among the priorities in improving the health of older adults.

**Electronic supplementary material:**

The online version of this article (10.1186/s12889-018-6288-6) contains supplementary material, which is available to authorized users.

## Background

Social and economic resources shape the health of individuals and populations. Richer countries tend to have better average health than poorer ones [1], and, within richer countries, lower socioeconomic status (SES) is associated with unhealthy lifestyle behaviors and higher morbidity, disability and mortality rates [2]. Education and financial status are usually considered as the most basic SES components.

The novelty of the present work is in defining and understanding health as not only the absence of disease, but as a vector of functioning in a sparing set of domains that matches the intuitive notion of health such that the health of people with distinct health problems or diseases could be compared, using a previously developed and validated health metric [3, 4]. Following the World Health Organization (WHO), health is understood as: (i) an intrinsic attribute of an individual that can be aggregated to the population level; and (ii) comprising domains of human functioning that describe the actual impact of health conditions on people’s lives [5].

The past decades, healthy aging was identified as the ability to detach oneself from the activities of mid-life as a kind of a preparatory step towards death and the aging process was assumed as a progressive, linear recession towards death [6]. Nowadays, healthy aging is described by the WHO as a process of developing and maintaining the functional ability that enables well-being in older age [7]. Within the Aging Trajectories of Health: Longitudinal Opportunities and Synergies (ATHLOS) project (EU HORIZON2020–PHC-635316, http://athlosproject.eu/) [4] the aim of the present work was to longitudinally explore the association between education and wealth status on 10-year all-cause mortality among the English Longitudinal Study of Aging (ELSA) participants, in relation to parameters of healthy aging, i.e., impairments in body functions and cognitive performance. Lifestyle behaviors, such as physical activity, alcohol consumption and smoking, were also evaluated as potential mediating pathways in the association between the socioeconomic determinants and healthy aging.

## Methods

Data from a national and representative study of the English population, i.e., the English Longitudinal Study of Aging (ELSA) [8], were used in this work. ELSA is large-scale, panel study of 12,099 participants, aged ≥50 years, living in England, and is one of the longitudinal studies included in the ATHLOS project (an EU/HORIZON2020 funded project that aims to identify health trajectories and determinants of aging). Study’s participants were re-examined during the study’s course (2002–2012) in six-periodic examinations (waves), every 2 years, i.e., in 2004 (wave 2), 2006 (wave 3), 2008 (wave 4), 2010 (wave 5) and 2012 (wave 6). The methods of the ELSA study mentioned in the present work have also previously been described in detail [8].

All participants have given written informed consent. Ethical approval for all the ELSA waves was granted from the National Research Ethics Service (MREC/01/2/91). Details of the ELSA study design, sample and data collection are available at the ELSA’s project website [https://www.elsa-project.ac.uk/].

From the initial sample of 12,099 participants at baseline evaluation, 175 subjects (1.45%) were excluded from the analysis since they were unable to be interviewed due to poor health. Moreover, 18 subjects (0.15%) were also excluded since their information was missing for at least the 25% of the health questions and measured tests at baseline examination and, 1000 participants were excluded because they reported “foreign/other” highest educational qualification and, thus, could not be classified in one of the educational levels generated for the purposes of the present work. The working sample was comprised of 10,906 participants (4967 men (64 ± 10 years) and 5939 women (64 ± 12 years)) who participated in the ELSA baseline examination. No differences as regards, age and sex distributions were observed between the initial and the working sample.

### Socio-demographic and lifestyle measurements

Socio-demographic measurements retrieved from ELSA database and used in this work were: sex (men/women), age (in years), formal household wealth and educational qualification. Specifically, according to the ELSA study’s protocol, wealth status refer to household wealth including financial, physical, and housing wealth, but not pension wealth. Wealth was calculated as net of debt and included the value of any home and other property (fewer mortgages); financial assets covering all types of savings available in England; the value of any business assets and physical wealth, such as artwork and jewellery. For the purposes of the present work, participants were classified into 3 classes of household wealth: Low (1st–2nd quintile, *n* = 3980, 39%), Moderate (3rd quintile, *n* = 2002, 20%) and High (4th–5th quintile, *n* = 4082, 41%). For the educational status, participants were classified into three groups based on the highest qualification achieved, i.e.,: Low status, indicating that the individual left education without any formal qualifications (i.e., 0–12 years of education – compulsory education) or after only completing National Vocational Qualifications (NVQs) at level 1 (*n* = 5488, 50%); Moderate status, which included participants who had completed high school (i.e., 12–14 years of education) or equivalent qualifications (O-level, A-level, or National Vocational Qualifications [NVQs] at levels 2–3) (*n* = 2710, 25%); and High status, including those participants with college or university degrees or NVQ at Level 4 or 5 (i.e., 15+ years of education) (*n* = 2688, 25%). Education and wealth status were assessed at baseline, as well as at each of the six waves.

Participants were also asked how often they took part in vigorous-intensity (e.g., running/jogging, swimming, cycling, aerobics/gym workout, tennis, and digging with a spade), moderate-intensity (gardening, cleaning the car, walking at moderate pace, dancing) and low-intensity (laundry and home repairs) physical activities, using prompt cards with different activities to help them interpret different activity intensities. Response options were: more than once a week, once a week, one to three times a month, and hardly ever/never. At each time point, physical activity was further categorized into 3 categories: Inactive; only light activity at least once a week (but no moderate or vigorous) (*n* = 920, 8%); Moderate activity at least once a week (but no vigorous) (*n* = 1155, 11%), and Vigorous activity at least once a week (*n* = 8829, 81%). These thresholds were chosen based on previous work in ELSA demonstrating robust dose-response associations with mortality [9]. According to their smoking habits, participants were classified as never smokers (*n* = 3890, 36%), former smokers (i.e., those who had quitted smoking before their enrollment in the study, *n* = 5017, 46%) and current smokers (i.e, those who were still smoking during their enrollment in the study, *n* = 1998, 18%). Moreover, the frequency of any alcohol consumed in the past 12 months was recorded and responses varied from 1 “almost every day” to 8 “not at all in the past 12 months” and were classified into 3 groups: “Never” (*n* = 1278, 12%), “Twice a week or less” (*n* = 6537, 60%) and “More than twice a week” (*n* = 3087, 28%).

### A metric of health status across all ELSA waves

To answer the research question of the present work, i.e., whether the effect of education and wealth status on all-cause mortality is mediated by aging factors, a health metric, as a proxy of healthy aging, has been used. In a previous publication of the ATHLOS project a health metric that incorporated factors associated with aging process, has already been introduced and validated [4]. Briefly, a set of 45 items were identified, comprising: 39 self-reported health questions related to impairments in body functions, limitations in Activities of Daily Living (ADL), and limitations in Instrumental Activities of Daily Living (IADL), and the other six variables were a set of tests covering cognitive functioning and walking speed [4]. This health metric has been calculated for each one of the six ELSA waves. The theoretical range of the health metric was from 0 to 100; higher values in the health metric score are indicative of better health status. The Health metric score descriptives (i.e., mean ± sd and median (range)) of the ELSA project participants across the six ELSA waves are presented in Additional file 1: Appendix 1.

### 10-year follow-up (2002–2012)

All-cause deaths were recorded. The time of death was available only by year, thus, binary time-specific event indicators were created for each study period. However, for *n* = 358 participants although the status of death was available, time of death was unknown, thus, information was retrieved looking whether respondents took part in the following surveys; if they were interviewed in later waves, they were assumed to be alive at least until the year of the last survey they responded, otherwise they were considered lost to follow-up. Thus, the participants were characterized as: (1) survivors or censored: if they did not experience a fatal event and were followed-up for all time-periods; (2) dead: if they had a fatal event during the study period; or (3) lost to follow-up: drop-outs. Mortality status was updated at February 2012. using the National Health Service Central Register, based on the informed consent provided by the participants at baseline.

### Statistical analysis

**Fig. 1:**
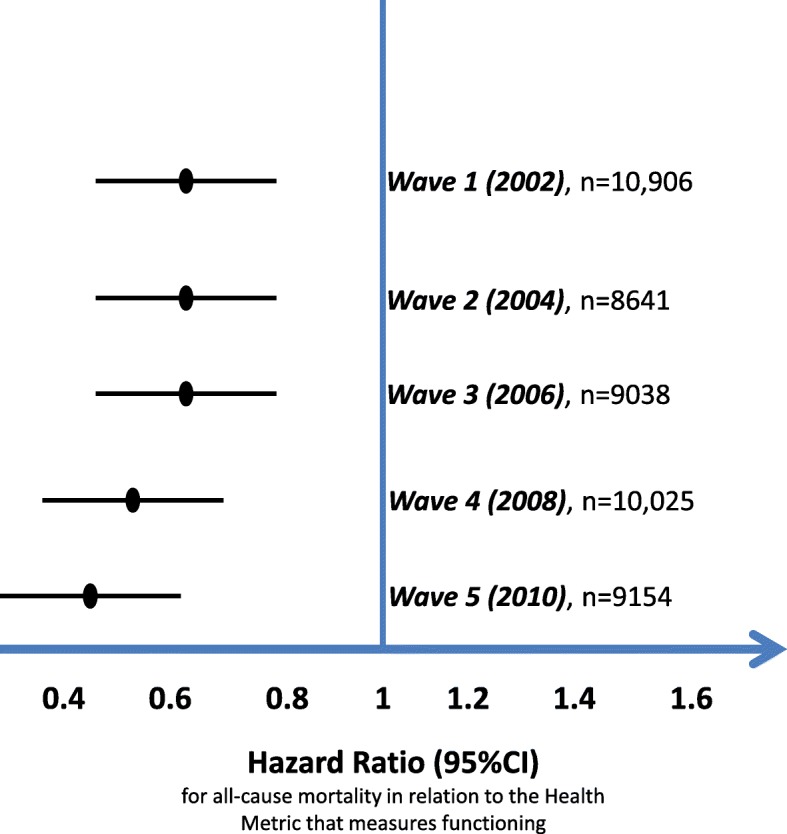
10-year mortality hazard ratios (per 10 unit increment) and 95% confidence intervals in relation to the health metric (higher values indicate better health status) that evaluated functioning characteristics of the ELSA participants, at the five waves (adjusted for age, gender, education and household wealth status)

**Fig. 2:**
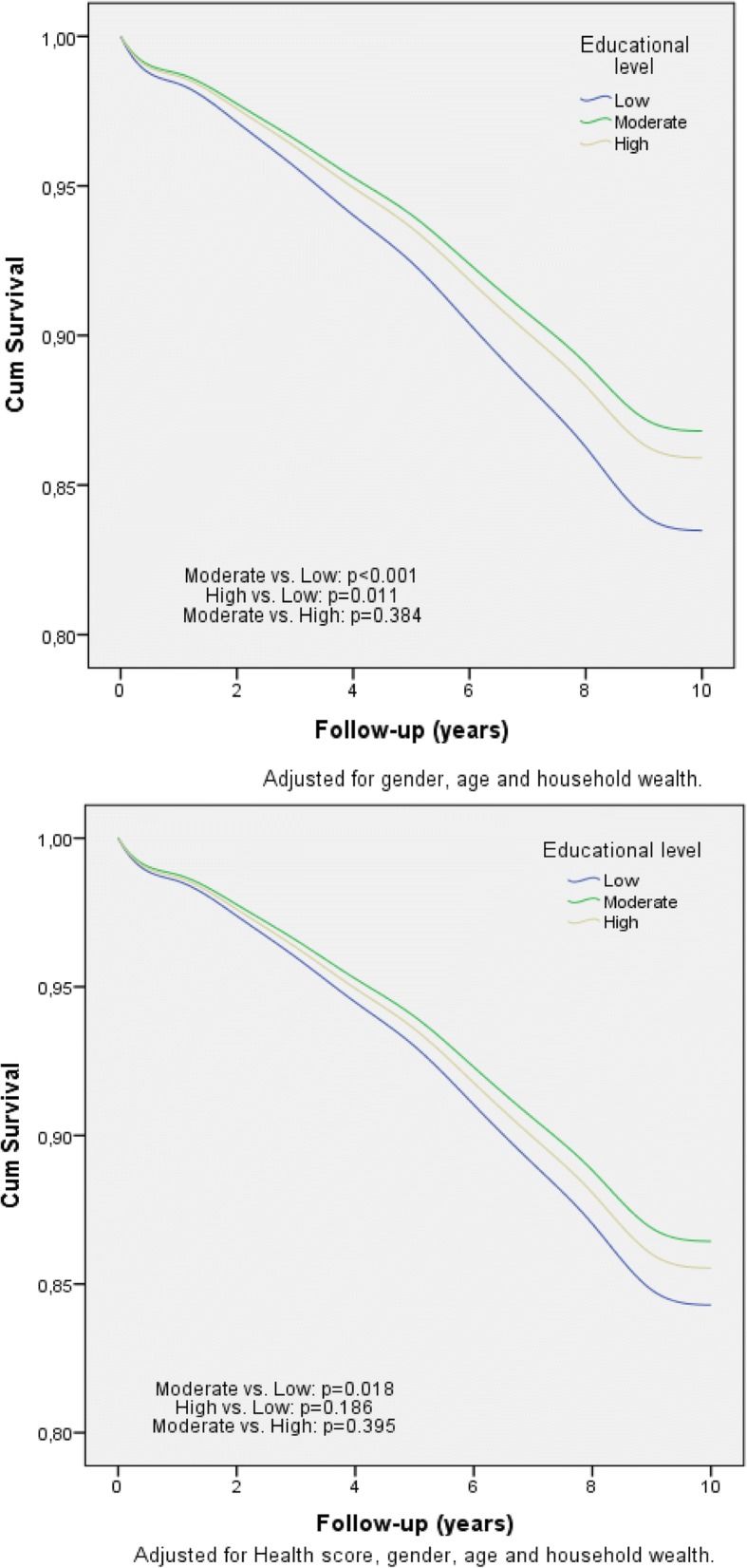
Kaplan–Meier survival plots, by educational level and household wealth class of the ELSA project participants (*n* = 10,906)

The unadjusted associations were assessed by applying t-test, ANOVA and Pearson r correlation where appropriate. In order to assess the over time age/gender-adjusted effect of educational level and household wealth on the health metric calculated at each one of the 6 ELSA waves, a mixed-effects, multilevel regression was conducted using data from all of waves (specified as levels and included in the model as random effects). Then, smoking habits, physical activity and alcohol consumption, were introduced into the model, to assess their potential mediating effect on the education-health and wealth-health associations. Sobel’s test was applied to test the significance of the mediation hypothesis [10]. Furthermore, Cox proportional hazard models assessed the associations of health metric, household wealth and educational qualification on the 10-year mortality rate. The proportionality assumption was assessed graphically and the estimated mortality hazard ratios (HRs) per 10 units increase in the health metric, adjusted for age, sex, education and household wealth for each of the ELSA waves were presented in Fig. 1. Moreover, the corresponding 10-year survival curves were constructed (i.e., according to the educational and household wealth level recorded at baseline) and compared by applying the log-rank test (Fig. 2). All reported *p*-values were based on two-sided tests. STATA 14 (Stata Corp LLC, Texas, USA) software was used for the statistical calculations.

## Results

During the 10-year follow-up, 2285 deaths were recorded, 19% in women and 25% in men (*p* < 0.001). In general, demographic characteristics were quite similar between women and men [3, 8]. In Table 1 various baseline demographic, socioeconomic, lifestyle characteristics and the health metric (across all six waves) in relation to 10-year all-cause mortality are presented.

**Table 1:** Baseline characteristics of the ELSA project participants’ in relation to 10-year all-cause mortality status (*n* = 10,906)

		10-year all-cause mortality		
		Alive	Dead	*p*
Age at baseline	Years, mean ±SD^a^	61 ± 10	75 ± 10	< 0.001
Gender	Women, *n* (%)	4647 (81%)	1094 (19%)	< 0.001
	Men, *n* (%)	3635 (75%)	1191 (25%)	
Education qualification	Low, *n* (%)	3726 (45%)	1595 (70%)	< 0.001
	Moderate, *n* (%)	2303 (28%)	323 (14%)	
	High, *n* (%)	2249 (27%)	363 (16%)	
Household wealth	Low, *n* (%)	2658 (35%)	1213 (54%)	< 0.001
	Moderate, *n* (%)	1543 (21%)	403 (18%)	
	High, *n* (%)	3341 (44%)	630 (28%)	
Smoking habits	Never smoker, *n* (%)	3144 (38%)	616 (27%)	< 0.001
	Former smoker, *n* (%)	3661 (44%)	1213 (53%)	
	Current smoker, *n* (%)	1477 (18%)	455 (20%)	
Physical activity	Inactive, *n* (%)	412 (5%)	476 (21%)	< 0.001
	Moderate, *n* (%)	793 (10%)	332 (14%)	
	Vigorous, *n* (%)	7077 (85%)	1476 (65%)	
Alcohol consumption	Never, *n* (%)	780 (9%)	447 (20%)	< 0.001
	Twice a week or less, *n* (%)	5110 (62%)	1234 (54%)	
	More than twice a week, *n* (%)	2389 (29%)	603 (26%)	
Health metric score^b^ (0–100)	Wave 1 (2002), mean ± SD	70 ± 11	60 ± 16	< 0.001
	Wave 2 (2004), mean ± SD	70 ± 11	60 ± 16	< 0.001
	Wave 3 (2006), mean ± SD	68 ± 12	55 ± 17	< 0.001
	Wave 4 (2008), mean ± SD	65 ± 12	51 ± 16	< 0.001
	Wave 5 (2010), mean ± SD	68 ± 13	45 ± 19	< 0.001
	Wave 6 (2012), mean ± SD	65 ± 14	40 ± 38	0.014

^a^*SD* standard deviation, ^b^Higher values in the health metric score that evaluates functionality are indicative of better health status

### Education, household wealth, lifestyle behaviors and the health metric of functioning and cognition, across study’s course

The baseline mean health metric score was 69/100 ± 12 for men and 67/100 ± 13 for women (*p* < 0.001), while it was progressively reduced across study’s course (Table 1). Moreover, the health metric was inversely correlated with age of the participants across all six waves of the study (*r* values from − 0.35 to − 0.29, *p* < 0.001). After adjusting for sex and age, education and household wealth were associated with the health metric throughout the 10 years of follow-up (Table 2). In particular, compared to individuals with low formal education, the moderately and highly educated participants had higher health metric scores (*p* < 0.001); however, no differences were observed between moderate and high education classes as regards their effect on the health metric (*p* = 0.109). Similar trends were observed for household wealth where, considering low class as the reference category, the middle and the higher classes had on average higher health metric scores (*p* < 0.001); moreover, the highest household wealth class had increased health score compared to the moderate class (*p* < 0.001). After age and gender adjustments, individuals belonging both in the lowest education and lowest wealth classes, had much lower health score (*p* < 0.001) compared to each one of the rest combinations of these social determinants (data not shown).

**Table 2:** Mixed linear regression model results (b-coefficient and 95% confidence interval (CI)) evaluating the age/gender-adjusted effects of educational level and household wealth on the metric that used as a proxy of healthy aging, after taking into account the effect of lifestyle behaviors, of the *n* = 10,906 ELSA participants, across all ELSA study waves (2002–2012)

	Model 1	Model 2	Model 3	Model 4
	b (95% CI)	b (95% CI)	b (95% CI)	b (95% CI)
Educational level (Ref. Low)				
Moderate	2.00 (1.60, 2.40)	1.96 (1.55, 2.36)	1.68 (1.31, 2.05)	1.88 (1.48, 2.27)
High	2.64 (2.25, 3.03)	2.54 (2.15, 2.93)	2.28 (1.91, 2.64)	2.48 (2.09, 2.87)
Household wealth (Ref. Low)				
Moderate	1.94 (1.68, 2.20)	1.91 (1.65, 2.17)	1.82 (1.57, 2.07)	1.88 (1.62, 2.14)
High	3.10 (2.81, 3.39)	3.06 (2.77, 3.34)	2.90 (2.63, 3.17)	2.99 (2.70, 3.28)
Gender (Ref. Female)	1.93 (1.56, 2.29)	2.15 (1.79, 2.52)	2.09 (1.75, 2.42)	1.74 (1.38, 2.11)
Age (years)	−0.39 (−0.41, −0.37)	− 0.39 (− 0.40, − 0.37)	−0.33 (− 0.35, − 0.32)	−0.38 (− 0.39, − 0.36)
Smoking habits (Ref. Never smoker)				
Former smoker		−1.43 (− 1.76, − 1.11)		
Current smoker		−1.41 (− 1.87, − 0.95)		
Physical activity (Ref. Inactive)				
Moderate activity			6.12 (5.56, 6.68)	
Vigorous activity			8.85 (8.32, 9.38)	
Alcohol consumption (Ref. Never)				
Twice a week or less				1.67 (1.39, 1.94)
More than twice a week				2.36 (2.05, 2.66)

Indicators of lifestyle behaviors, such as smoking, physical activity and alcohol consumption were also associated with the health metric of functioning and cognition. In particular, the metric was inversely associated with smoking status (*p* < 0.001) (i.e., smokers had lower functioning and cognition abilities across aging) and positively associated with education (i.e., better education level, higher functioning and cognition across aging), household wealth (i.e., better wealth status higher functioning and cognition), physical activity level (i.e., engaging in physical activities higher functioning and cognition during aging) and alcohol consumption pattern (moderate alcohol drinking better functioning and cognition level) (all *p*-values < 0.001). The aforementioned lifestyle factors also acted as mediators in the education and wealth associations with the health metric (*p* < 0.001). All the three, reduced significantly (i.e., mediated) the effect size of both the educational and economic level on the health metric, which nevertheless remained significant. A linear, positive correlation was observed for both physical activity and alcohol consumption with the health metric, while smoking (currently or formerly) was associated with lower health scores (*p* < 0.001); current smokers did not differ from the former in terms of the health metric (*p* = 0.921). No significant interaction was observed between sex of the participants and educational level (*p* = 0.339) or household wealth (*p* = 0.116) in their relationship with the health metric.

### Education and household wealth as predictors of 10-year all-cause mortality: The mediating effect of the health metric of functioning and cognition

The health metric was inversely and progressively associated with 10-year all-cause mortality risk across the six waves (Fig. 1). Moreover, the age-gender adjusted all-cause mortality risk was 22% lower for the moderately educated (*p* < 0.001) and 16% lower for the highly educated (*p* = 0.011) as compared to the lowest education class; there was no difference between moderate and high education classes (*p* = 0.384). After further adjusting for the metric that evaluated healthy aging status of the participants, only the moderate educated class differed significantly compared to the lowest class as regards the 10-year mortality rate (*p* = 0.018). Sobel test yielded a significant mediating impact of the health metric on the observed education-mortality association (*p* = 0.02). Concerning household wealth, both the middle and highest classes had 26 and 35% reduced mortality risk as compared to the lowest class, respectively (*p* < 0.001), while the middle class compared to highest, had also increased mortality risk (14%, *p* = 0.057). As in the case of education level, the introduction of the health metric in the aforementioned model mediated the wealth-mortality association (*p* < 0.001) by reducing the effect size of wealth status on mortality (Sobel’s *p* = 0.01). However, the association of household wealth on mortality remained significant, even after adjusting for the health metric of functioning and cognition, with the highest and middle classes having lower risk as compared to the lowest class (*p* = 0.001); no differences between the middle and highest class were observed as regards their effect on mortality (*p* = 0.279). Moreover, no significant interactions were observed between gender of the participants and educational level (*p* = 0.09) or household wealth (*p* = 0.188) as regards 10-year mortality risk. The corresponding estimated survival curves are provided in Fig. 2. Similar were the results when analyzing education and wealth status from the rest of the ELSA waves (data not presented here).

## Discussion

The present study verified a strong association between two of the SES determinants (i.e., education and financial status) and 10-year mortality. Moreover, the health metric reflecting functionality [4] was identified as a strong and independent protective factor against 10-year mortality and actually with the passage of time, i.e., increasing age; it seems to be more effective in evaluating all-cause mortality. Furthermore, education and financial status over time were both proved as strong and independent predictors of healthy aging as it was estimated through the health metric score, while physical activity, smoking and alcohol consumption were identified as mediators in this relationship. Respondents with lower household wealth or education, had lower scores on the health metric which in turn led to higher mortality during the follow up through the adaptation of unhealthy lifestyle behaviors (i.e., smoking and physical inactivity) with the combination of low education and low wealth at the same time having the lowest health scores compared to all of the rest possible education-wealth combinations.

However, a linear positive association between education-household wealth and alcohol drinking was present while similar trends were found for the alcohol-health association. It has been observed that in the higher socioeconomic classes, low-risk drinking patterns are more common while the lower classes muster more alcohol abstainers [11]. However, it seems that alcohol consumption causes more adverse effects on low SES individuals. In our study, only the frequency of alcohol consumption was taken into account and not the volume, the quality or the pattern of drinking. Hence, the linear positive association between the frequency of alcohol consumption and health status could probably reflect “healthy” drinking patterns (e.g., drinking under social occasions and having a large social network). Additionally, for some individuals, the partial or total abstinence from alcohol could be due to an underlying poor health.

Additionally, although compared to non-smokers, former and current smokers had lower health scores as expected [12], the latter groups did not differ from each other in terms of the health metric. This could be possibly explained by the fact that current, ex- and passive smoking is associated with other risk factors, arising a potential confounding design [13]. Moreover, the reason for smoking cessation is often a non-reversible poor state of health.

Maintaining a relatively high level of health, functioning and well-being throughout life-course, could be seen as a precursor of longevity and lowered risk of chronic diseases [14–16]. In recent years, healthy aging has been addressed through a multidimensional approach, in order to capture and attribute its variability [15], with the individual’s functional ability and capacity being the key notions in the conceptualization of healthy aging [7].

However, the normal deterioration of health with aging can worsen under poor socioeconomic conditions. SES has been associated with a wide range of diseases, disabilities, risk factors, lower survival rates and cognitive performance, with the largest inequalities affecting middle-aged individuals [17, 18]. Several linking pathways in the SES-Health association have been proposed. Low SES classes have been linked to: i) unhealthy lifestyle behaviors [19], ii) poorer social network [11], iii) impaired environmental living conditions, iv) lower accessibility to health services [20], v) under-treatment [21] and vi) elevated chronic stress [22, 23].

Furthermore, the improvement in the management of chronic diseases, has not been able to delay declines in health with aging, leading to high cost for the society [24, 25]. Increased average life expectancy, combined with reduced fertility and population growth rates, is leading to higher old-age dependency ratios. Early preventive interventions, especially through lifestyle changes, can reduce the proportion of older people who have significant health decrements earlier on. Actually, healthy aging is already high on the European and global policy agendas [26]; however, effectively eliminating socioeconomic inequalities is a challenging but labored matter. It should be focused on all SES components and simultaneously take into account the SES-health linking mechanisms.

### Strengths and limitations

The fact that the reasons for the abstinence from alcohol and/or smoking were not recorded could be seen as a limitation of the present work, as a pure health state is frequently the cause and thus, it was not possible to distinguish between the abstainers of free choice and those who abstained after medical advice while the only available data on alcohol consumption was the 3-level distinction as follows: i) not drinking, ii) twice a week or less and iii) more than twice a week. Also, the exact years of educational attainment were not available in the ELSA survey and thus the corresponding educational levels were used. Additionally, there was not available information on the existence of several co-morbidities or drugs used that could probably have affected the results. Despite the aforementioned limitations, the use of a large and nationally representative sample of the English population coupled with the long follow-up with adequate reassessments (6 waves) makes it possible to draw safe and generalizable conclusions. Additionally, the employment of a latent score on health, which can be compared across longitudinal studies opens and facilitates the way for further research at multinational level.

## Conclusions

The present work contributes to the long discussion on the effect of social and lifestyle-related factors in health, as measured by functioning and cognition determinants, and in overall mortality, by showing that even among the less socially “privileged”, control of disabilities may lead to longer survival. In conclusion, it is evident that prevention actions should be targeted in limiting SES inequalities regarding health status and quality of life by focusing on the three most common social determinants, i.e., profession, income and education, and on the linking mechanisms between the latter and health. At least, as far as public health is concerned, socioeconomic disparities should be addressed thoroughly and implemental modifications should be made in favor of the most underprivileged societal classes. Given the pervasive effects of SES and in the light of the ongoing economic crisis in Europe, no single policy, or even one domain of policy, can eliminate health inequalities. Radical and coordinated interventions are required.

## Additional file

Additional file 1: **Appendix 1.** Table presenting the Health metric score descriptives (i.e., mean ± sd and median (range)) of the ELSA project participants across the six ELSA waves. (DOCX 15 kb)

## Abbreviations

ADL: Activities of Daily Living
ATHLOS: Aging Trajectories of Health: Longitudinal Opportunities and Synergies
CI: Confidence Intervals
ELSA: English Longitudinal Study of Aging
HR: Hazard Ratio
IADL: Instrumental Activities of Daily Living
NVQs: National Vocational Qualifications
SD: Standard Deviation
SES: Socioeconomic status
WHO: World Health Organization
