# Supplementary material for: Social determinants, health status and 10-year mortality among 10,906 older adults from the English longitudinal study of aging: the ATHLOS project
Source: BMC Public Health. 2018 Dec 10;18:1357. doi: 10.1186/s12889-018-6288-6 (PMC6288914; doi:10.1186/s12889-018-6288-6)
Supplement: Supplementary file 1 — Appendix 1. Table presenting the Health metric score descriptives (i.e., mean ± sd and median (range)) of the ELSA project participants across the six ELSA waves. (DOCX 15 kb) [file 12889_2018_6288_MOESM1_ESM.docx]

| **Appendix 1.** Health metric score descriptive of the ELSA project participants’ presented both as mean ± SD^a^ and median (range) (*n*=10,906). | | | |
| --- | --- | --- | --- |
|  |  | mean±SD | median (range) |
| Health metric score^b^ (0-100) | Wave 1 (2002) | 68±13 | 74 (8–83) |
|  | Wave 2 (2004) | 69±13 | 74 (6–96) |
|  | Wave 3 (2006) | 67±13 | 73 (9–97) |
|  | Wave 4 (2008) | 66±13 | 71 (3–85) |
|  | Wave 5 (2010) | 69±13 | 75 (11–100) |
|  | Wave 6 (2012) | 67±13 | 72 (7–97) |
| ^a^ SD: standard deviation; ^b^ Higher values in the health metric score that evaluates functionality are indicative of better health status. | | | |
